# Ethnic and social disparities in different types of examinations in undergraduate pre-clinical training

**DOI:** 10.1007/s10459-016-9676-7

**Published:** 2016-03-25

**Authors:** K. M. Stegers-Jager, F. N. Brommet, A. P. N. Themmen

**Affiliations:** 1 Institute of Medical Education Research Rotterdam, Erasmus MC, University Medical Centre Rotterdam, Room AE-241, PO Box 2040, 3000 CA Rotterdam, Netherlands; 2 Department of Internal Medicine, Erasmus MC, University Medical Centre Rotterdam, Rotterdam, Netherlands

**Keywords:** Assessment, Ethnic(ity), Gender, Language skills, Logistic regression, Medical students, Performance, Social background, Undergraduate

## Abstract

Medical schools are increasingly faced with a more diverse student population. Generally, ethnic minority students are reported to underperform compared with those from the ethnic majority. However, there are inconsistencies in findings in different types of examinations. Additionally, little is known about the performance of first-generation university students and about performance differences across ethnic minority groups. This study aimed to investigate underperformance across ethnic minority groups and by first-generation university students in different types of written tests and clinical skills examinations during pre-clinical training. A longitudinal prospective cohort study of progress on a 3-year Dutch Bachelor of Medicine course was conducted. Participants included 2432 students who entered the course over a consecutive 6-year period (2008–2013). Compared with Dutch students, the three non-Western ethnic minority groups (Turkish/Moroccan/African, Surinamese/Antillean and Asian) underperformed in the clinical problem solving tests, the language test and the OSCEs. Findings on the theoretical end-of-block tests and writing skills tests, and results for Western minority students were less consistent. Age, gender, pre-university grade point average and additional socio-demographic variables (including first-generation university student, first language, and medical doctor parent) could explain the ethnicity-related differences in theoretical examinations, but not in language, clinical and writing skills examinations. First-generation university students only underperformed in the language test. Apparently, underperformance differs both across ethnic subgroups and between different types of written and clinical examinations. Medical schools should ensure their assessment strategies create a level playing field for all students and explore reasons for underperformance in the clinical and writing skills examinations.

## Introduction

The past few decades have shown an increase in the number of students from non-traditional backgrounds, such as ethnic minority and first-generation university students, who enter medical school in Western countries (Arulampalam et al. 2004; Bedi and Gilthorpe 2000; Howe et al. 2004; Klimidis et al. 1997). The increase in non-traditional students is not only a result of changing demographics, but is also caused by widening access policies that medical schools have adopted to achieve social equality and to ensure that the population of matriculating medical doctors is more representative of society (Cleland et al. 2012). In general, students from ethnic minorities are reported to underperform compared with those from the ethnic majority. However, there are inconsistencies in findings in different types of examinations. In addition, little is known about the performance of first-generation university students and about performance differences across ethnic minority groups. In this study, we investigate underperformance across ethnic minority groups and by first-generation university students in different types of written and clinical skills examinations during pre-clinical training.

Ethnic minority students have been shown to underperform compared with those from the ethnic majority at different stages of medical school. Studies from Australia and the Netherlands show that they underperform in the first year of medical school (Kay-Lambkin et al. 2002; Stegers-Jager et al. 2012) and also later in the course, in final year assessments in Australia, the UK and the USA (Liddell and Koritsas 2004; McManus et al. 1996; Xu et al. 1993). A systematic review and meta-analysis showed that ethnic minority students in the UK academically underperform compared with white students throughout medical school and across different types of examinations (Woolf et al. 2011).

In particular differences in clinical performance between white students and ethnic minority students appear to be consistent: research from numerous Western medical schools has shown that ethnic minority students underperform compared with their white counterparts in Objective Structured Clinical Examinations (OSCEs) and other clinical assessments (Dewhurst et al. 2007; Haq et al. 2005; Lumb and Vail 2004; Woolf et al. 2008, 2013; Yates and James 2007). Others found that they received lower grades in their clerkships (Lee et al. 2007; Stegers-Jager et al. 2012). The underperformance in clinical assessments of ethnic minority students was also found among physiotherapy students (Naylor et al. 2014).

However, ethnic differences on performance in written examinations are less clear. Several authors have reported that ethnic disparities in the pre-clinical course were less profound than in the clinical course (Stegers-Jager et al. 2012; Yates and James 2007). Woolf et al. (2008) found ethnic differences on both written and OSCE assessments, but the difference on the written assessments disappeared when it was adjusted for OSCE performance, while it remained on the OSCE when adjusted for written performance. Haq et al. (2005) found ethnic differences on all OSCEs, but only on half of the written examinations.

In addition, to our knowledge, little is known about differences in performance on various types of written examinations, such as theoretical end-of-block tests, clinical problem solving tests and writing skills tests. Despite the fact that studies on ethnicity and medical school performance have used several types of written outcomes (Woolf et al. 2011), we are not aware of studies that have specifically looked at differences on performance in different types of written examinations.

Although traditionally medical students come from the highest socio-economic groups (Seyan et al. 2004), the anticipated effect of the widening access policies is an increase of so-called first-generation university medical students. The evidence with respect to the relationship between social background and medical school performance is inconclusive: some studies report that social-class background and parental education are not associated with performance of medical students in the UK and the USA (Arulampalam et al. 2004; Fernandez et al. 2007; Lumb and Vail 2004), whereas other studies report that students of lower social-class and first-generation university students underperform in medical school in the Netherlands and the UK, in particular in clinical examinations (Stegers-Jager et al. 2012; Woolf et al. 2013). Two studies report that students with a medical doctor as parent are less likely to drop out of medical school (Arulampalam et al. 2004, 2007), whereas this predictor was not confirmed in another study (Stegers-Jager et al. 2012). Irrespective of these reports there appears to be a relative paucity in the number of studies that have focused on social background as predictor of performance in medical school (O’Neill et al. 2011).

In sum, there is accumulating evidence of ethnic disparities and some of social disparities in preclinical training. However, it is still not clear why it occurs. The aim of this study was to gain more insight in reasons for underperformance by looking at performance in various types of written and clinical examinations. In addition, we examined whether the ethnic and social disparities in examination performance could be explained by a combination of socio-demographic and academic factors that have previously been found to influence medical school performance, including gender (Haq et al. 2005; James and Chilvers 2001; Lumb and Vail 2004; Yates and James 2007), age (James and Chilvers 2001; Lumb and Vail 2004; Stegers-Jager et al. 2012), first language (Arulampalam et al. 2007; Ferguson et al. 2002; McManus et al. 1996), and pre-university grades (Arulampalam et al. 2004; Huff and Fang 1999; Yates and James 2007). Finally, in our analysis we have also taken into account the reported variation in academic performance among different ethnic minority groups (Hofman and van den Berg 2003; McManus et al. 1996; Stegers-Jager et al. 2012).

## Methods

### Course structure and examination

This study was conducted at the Erasmus MC Medical School, Rotterdam, the Netherlands. Compared with other Dutch medical schools this school has a relatively large number of ethnic minority students. The medical course consists of a 3-year Bachelor degree course followed by a 3-year Masters degree course. The integrated and theme-oriented Bachelor curriculum is divided into three thematic blocks per year. Each thematic block consists of 2–3 sub-blocks. The Bachelor course includes two types of examinations: written examinations and clinical skills examinations. There are three types of written examinations: (1) theoretical knowledge, (2) language skills, and (3) writing skills. The theoretical knowledge examinations are further divided into block tests at the end of each thematic sub-block and a clinical problem solving test (CPST) at the end of each year of the Bachelor degree course. The theoretical examinations are largely machine-marked and consist of multiple choice questions (MCQs), extended matching questions (EMQs), comprehensive integrative puzzles (CIPs) and/or short answer questions. The language skills test consists of four parts: basic Dutch, spelling, grammar and style. The writing skills tests include writing an abstract (Year 1), an argument paper (Year 2) and an essay (Year 3).

### Participants and procedure

The new Erasmus MC Bachelor curriculum was implemented in 2008. All 2432 students who entered the Erasmus MC Medical School during 2008–2013 were included in this study. Data on ethnicity, gender, age and pre-university Grade Point Average (pu-GPA) for these cohorts were available from a national database of students in higher education in the Netherlands (1cijferHO).

Additional data on social background were collected by online questionnaire for first-year students in 2012 (n = 331; 83 %) and in 2013 (n = 392; 95 %) and for third-year students in 2012 (n = 340; 92 %). This part of the study was designed with the help and approval of the Dutch Data Protection Authority. Students were informed about the study, participation was voluntary, confidential processing was guaranteed, and individual consent was sought. Data on examination performance were obtained from the university student administration system. Because these data were collected as part of regular academic activities, individual consent was not necessary.

### Variables and measures

The socio-demographic variables included in this study are ethnicity, first-generation immigrant, urban background, first-generation university student, first language, and medical doctor as parent.

According to Statistics Netherlands (CBS; www.cbs.nl), an individual belongs to an ethnic minority group if at least one of his or her parents was born outside the Netherlands. Based on the countries of birth of their parents, ethnic minority students were classified into one of five ethnic subgroups: Turkish/Moroccan/African; Surinamese/Antillean; Asian (mainly Middle East: Afghanistan, Iran, Iraq, Pakistan); Western, and ‘Other’ (Hofman and van den Berg 2003; Stegers-Jager et al. 2012). Because of the small number of students in the category ‘Other’ (n = 19), their data were excluded from the analyses.

On the questionnaire, students self-categorized whether they had an urban background and whether their first language was Dutch or non-Dutch. Parental education and parental profession as provided by the students was used to determine whether or not they were first-generation university students and whether or not they had at least one parent who was a medical doctor.

As confounders we included gender, age and pu-GPA. The age at entry of medical school was split into three categories: <19 years; 19–21 years; >21 years. pu-GPA was included in the analyses as a continuous variable. As pu-GPA was not available for students with a foreign or a non-standard Dutch pre-university education, a categorical variable—‘missing pu-GPA’—was added to the analyses. Missing values for pu-GPA were substituted with the mean in an analysis with ‘missing pu-GPA’ and continuous pu-GPA included.

Outcome measures were ‘pass’ or ‘fail’ on the written and clinical examinations. The cut-off pass/fail mark was 5.5 on a 10-point scale (1 = poor, 10 = excellent). Two first-year end-of-block tests and the three end-of-year CPSTs were included in this study. We also included the first-year language skills test and the three writing skills tests (one each year). Finally, the clinical skills examinations in year 2 and 3 of the bachelor degree course were included in this study. The second-year OSCE consists of three stations: history taking, physical examination and communication. The third-year OSCE consists of seven stations: history taking (2×), physical examination (2×), communication (2×) and neurology (1×). Measures of reliability of the written and clinical examinations were generally >0.7. Only data of the first attempt on the examinations were included. The number of participants per examination per cohort differs because of absence at examinations and, in particularly in later years, of attrition, either voluntarily or due to dismissal (see Stegers-Jager et al. 2011 for more details).

### Statistical analysis

We assessed associations between ethnicity and the other independent variables using Chi squared tests for categorical data and analysis of variance (ANOVA) for pu-GPA. We used logistic regression to calculate odds ratios (ORs) for the effect of ethnicity on the outcome measures (Step 1). Subsequently, we adjusted for key confounders (gender, age, and pu-GPA) (Step 2). Finally, we adjusted for confounders and socio-demographic characteristics (first-generation immigrant, urban background, first language, medical doctor as parent and first-generation university student) (Step 3).

Similar analyses were used to calculate the effect of being a first-generation university student.

In order to obtain an indication of the effect of ethnicity per type of exam we took a meta-analytic approach. For each of the ethnic minority subgroups, we pooled odds ratios using mixed effects logistic regression for each type of examination and for all examinations combined. These meta-analyses were performed on both the unadjusted (Step 1) and the adjusted (Step 3) odds ratios. The glmer command in R was used to estimate the logistic regression models with ethnicity (unadjusted and adjusted), confounders and socio-demographic characteristics as predictors and pass/fail on the various examinations as outcomes.

Missing values on ‘first-generation university student’, ‘medical doctor as parent’, ‘first language’ and ‘urban background’ were statistically imputed based on their correlation with the other variables, including the outcome variables (Appendix 2). We used the multiple imputations procedure in SPSS, and chose to replace each missing value five times using five independent draws from the imputation model. The multiple imputation for categorical variables was restricted so that only categorical imputations were produced. Pooled estimates over the imputed data sets were used. As recommended by Sterne et al. (2009) odds ratios were compared between analyses of the imputed dataset (multiple imputed) and the unimputed dataset (complete cases) (see Appendix 2 for details). As the absence of data on the four imputed variables was systematically related to cohort, we considered the missing-at-random assumption to be reasonable.

Meta-analyses were performed using R statistical software, version 3.1.0 (R Foundation for Statistical Computing, Vienna, Austria), all other statistical analyses were performed using IBM SPSS Statistics for Windows Version 21.0 (IBM Corp., Armonk, NY, USA). We present 95 % confidence intervals (CIs) for adjusted ORs, which indicate statistical significance if they do not include a value of 1.0.

## Results

### Student characteristics

Non-Dutch students on average were older, more often had a missing pu-GPA and an urban background (Table 1). Turkish/Moroccan/African students were more often first-generation university students and had less often a medical doctor as parent. Asian students were more often male. Non-Dutch as a first language was more often spoken among Turkish/Moroccan/African and Asian students. The mean pu-GPA of Turkish/Moroccan/African students and Asian students was significantly lower than of Dutch and Western students. There was no statistically significant difference in the numbers of students in each ethnic category between the six cohorts.

**Table 1:** Characteristics of 2413 students in the cohorts of 2008–2013

	2008–2013 (n = 2413^a^)^b^												
	Dutch (n = 1645, 68 %)		Turkish/Moroccan/African (n = 197, 8 %)		Surinamese/Antillean (n = 155, 6 %)		Asian (n = 212, 9 %)		Western (n = 204, 9 %)		Total		*p* value
	n	%	n	%	n	%	n	%	n	%	n	%	
*Confounders*													
Male	604	**37**	85	43	67	43	96	45^b^	74	36	926	38	0.04
Age													<0.001
<19	949	**57**	80	**41**	78	50	74	**35**	99	49	1280	53	
19–21	535	**33**	91	**46**	53	34	97	**46**	70	34	846	35	
>21	161	**10**	26	13	24	16	41	**19**	35	**17**	287	12	
Pre-university GPA													
Missing^d^	128	**8**	33	17	66	**43**	38	**18**	39	**19**	304	13	<0.001
Mean (SD) (n = 2109)	7.15 (0.61)		6.91 (0.59)^c^		7.01 (0.53)		7.00 (0.63)^c^		7.18 (0.60)				<0.001
Cohort													0.44
*Socio*-*demographic characteristics*													
First-generation immigrant	–	–	21	**11**	48	31	121	**57**	53	**26**	243	32	<0.001^e^
First language non-Dutch (n = 1052)	*11*	*2*	60	**78**	15	**28**	78	**89**	33	**39**	186	61	<0.001^e^
First-generation university student (n = 1048)	162	**22**	41	**55**	14	27	28	32.	13	**16**	258	25	<0.001
Medical doctor as parent (n = 1047)	97	13	3	**4**	8	15	7	8.	15	18	130	12	0.05
Urban background (n = 1050)	376	**50**	71	**92**	39	**75**	63	**72**	53	62	602	57	<0.001

*GPA* grade point average, *SD* standard deviation

^a^19 students in the category ‘other’ were excluded from analyses

^b^Figures in bold denote percentages significantly different from overall average (*p* < 0.05)

^c^Pre-university GPA significantly lower than for Dutch and Western students

^d^Number of students with a foreign pre-university education: Dutch, n = 16; Turkish/Moroccan/African, n = 4; Surinamese/Antillean, n = 31; Asian, n = 7; Western, n = 20

^e^Category Dutch excluded from analysis

### Written examinations: theoretical knowledge

**Fig. 1:**
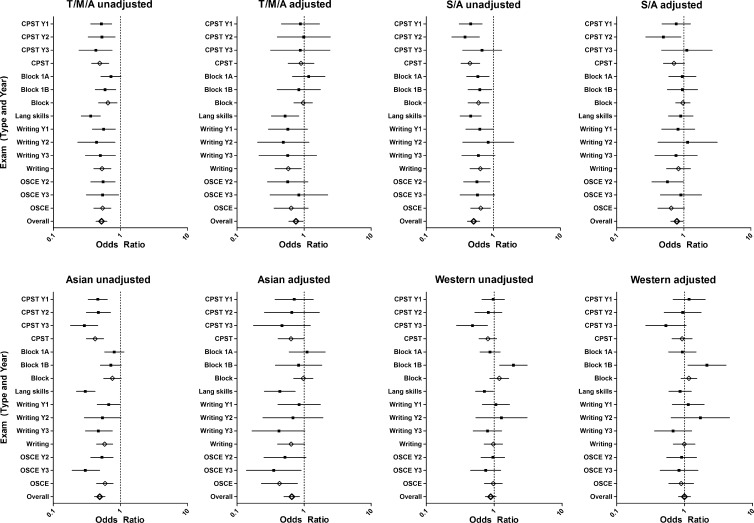
Unadjusted and adjusted odds ratios and 95 % confidence intervals for the effect of ethnicity on performance in written and clinical examinations; Dutch as reference group. *Black boxes* represent odds ratios for individual examinations; *diamonds* represent the pooled odds ratios for examination types and all examinations combined. *T/M/A* Turkish/Moroccan/African; *S/A* Surinamese/Antillean, *CPST* Clinical Problem Solving Test, *Lang* language, *OSCE* Objective Structured Clinical Examination, *Y* year. Adjusted refers to: adjusted for age, gender and pre-university grade point average (confounders), cohort and first-generation immigrant, urban background, first language non-Dutch, medical doctor as parent and first generation university (socio-demographic characteristics)

Dutch students were more likely to pass the first-year CPST (74 %) compared with Turkish/Moroccan/African students (60 %; unadjusted OR 0.52), Surinamese/Antillean students (57 %; unadjusted OR 0.46) and Asian students (57 %; unadjusted OR 0.46; Table 2 and Appendix 1, Fig. 1). Similar results were found for the second-year CPST (see Table 2 and Appendix 1; Fig. 1). On the third-year CPST Dutch students (85 %) were more likely to pass compared with Turkish/Moroccan/African students (71 %; unadjusted OR 0.43) and Asian students (62 %; unadjusted OR 0.29). Dutch students were also more likely to pass first-year block test A (68 %) compared with Surinamese/Antillean students (55 %; unadjusted OR 0.59). Turkish/Moroccan/African and Surinamese/Antillean students were less likely to pass first-year block test B compared with Dutch students (65 and 67 %, respectively vs 76 %; unadjusted ORs 0.59 and 0.63), while Western students were more likely than Dutch students to pass this test (86 %; unadjusted OR 1.92). All these disparities were to a large extent explained by confounders and socio-demographic characteristics (Fig. 1). Details of the regression analyses, with both complete cases and multiple imputations, are presented in Appendix 2.

**Table 2:** Pass/fail rates on the theoretical, language, writing skills and clinical skills examinations by ethnicity

	2008–2013 (n = 2413^a^)												
	Dutch (n = 1645, 68 %)		Turkish/Moroccan/African (n = 197, 8 %)		Surinamese/Antillean (n = 155, 6 %)		Asian (n = 212, 9 %)		Western (n = 204, 8 %)		Total		*p* value
	n	%	n	%	n	%	n	%	n	%	n	%	
*Theoretical knowledge*													
Passed CPST year 1 (n = 1837)	946	**74**	85	**60**	63	**57**	89	**57**	111	74	1294	70	<0.001
Passed CPST year 2 (n = 1317)	732	**80**	67	**68**	49	**61**	73	**66**	91	77	1012	77	<0.001
Passed CPST year 3 (n = 974)	572	**85**	48	**71**	46	79	53	**62**	64	73	783	80	<0.001
Passed block test year 1 A (n = 1902)	885	**68**	91	60	65	**55**	102	63	101	64	1244	65	0.030
Passed block test year 1 B (n = 1884)	986	76	98	**65**	78	**67**	114	70	134	**86**	1410	75	<0.001
*Language skills*													
Passed language skills test year 1 (n = 2233)	864	**56**	56	**32**	53	**37**	54	**28**	91	48	1118	50	<0.001
*Writing skills*													
Passed writing skills test year 1 (n = 1519)	814	**77**	78	**65**	58	67	93	69	91	78	1134	75	0.009
Passed writing skills test year 2 (n = 1022)	650	91	62	**82**	51	90	77	85	78	93	918	90	0.035
Passed writing skills test year 3 (n = 949)	478	**73**	37	**57**	35	61	45	**56**	59	68	654	69	0.002
*Clinical skills*													
Passed OSCE year 2 (n = 1379)	671	**71**	60	**58**	49	**58**	68	**57**	90	70	938	68	<0.001
Passed OSCE year 3 (n = 962)	530	**79**	43	67	40	69	44	**54**	66	74	723	75	<0.001

*CPST* Clinical Problem Solving Test, *OSCE* Objective Structured Clinical Examination

Figures in bold denote percentages significantly different from overall average (*p* < 0.05)

^a^19 students in the category ‘other’ were excluded from analyses

The pass/fail rates between first-generation university students and non-first-generation university students were not significantly different on any of the theoretical knowledge tests (Table 3).

**Table 3:** Pass/fail rates on the theoretical, language, writing skills and clinical skills examinations by social background

	2008–2013 (n = 1048)				
	First-generation university (n = 258, 25 %)		Non-first-generation university (n = 790, 75 %)		*p* value
	n	%	n	%	
*Theoretical knowledge*					
Passed CPST year 1 (n = 633)	113	72	343	72	0.90
Passed CPST year 2 (n = 329)	74	83	191	80	0.47
Passed CPST year 3 (n = 325)	76	88	211	88	0.98
Passed block test 1 A (n = 647)	113	70	342	70	0.97
Passed block test 1 B (n = 642)	130	81	390	81	0.93
*Language skills*					
Passed language skills test (n = 966)	104	44	385	53	0.02
*Writing skills*					
Writing skills test year 1 (n = 642)	117	74	356	74	0.98
Writing skills test year 2 (n = 323)	79	91	217	92	0.74
Writing skills test year 3 (n = 314)	56	68	166	72	0.58
*Clinical skills*					
OSCE year 2 (n = 333)	66	74	192	79	0.38
OSCE year 3 (n = 322)	65	77	193	81	0.33

*CPST* Clinical Problem Solving Test, *OSCE* Objective Structured Clinical Examination

### Written examinations: language skills

The percentage of Dutch students that passed the language skills test (56 %) was significantly higher than for all other ethnic subgroups (ranging from 28 to 48 %; Table 2). The differences in percentages correspond to unadjusted ORs ranging from 0.30 for Asian students to 0.48 for Western students. These disparities were only partly explained by confounders and socio-demographic characteristics (Fig. 1; Appendix 1).

First-generation university students less often passed the language skills examination than non-first-generation university students (44 vs 53 %; Table 3). This difference corresponds to an unadjusted OR of 0.72 (95 % CI 0.56–0.93), which could largely be explained by confounders [adjusted OR 0.81 (95 % CI 0.63–1.05)] and socio-demographic factors [adjusted OR 0.91 (95 % CI 0.69–1.19)]. Results for the complete cases analysis were similar [unadjusted OR of 0.70 (95 % CI 0.52–0.94), and adjusted ORs of 0.78 (95 % CI 0.57–1.08) and of 0.79 (95 % CI 0.56–1.11), respectively].

### Written examinations: writing skills

Dutch students were more likely to pass the first-year writing skills test compared with Turkish/Moroccan/African students (77 vs 65 %; unadjusted OR 0.56) and Asian students (69 %; unadjusted OR 0.67). Similar results were found for the second and third-year writing skills tests (Table 3, Fig. 1). Confounders and socio-demographic characteristics could only partly explain these differences (Fig. 1, Appendix 1).

The pass/fail rate between first-generation university students and non-first-generation university students was not significantly different on the writing skills tests (Table 3).

### Clinical skills

Dutch students were more likely to pass the second-year OSCE (71 %) than Turkish/Moroccan/African students (58 %; unadjusted OR 0.55), Surinamese/Antillean students (58 %; unadjusted OR 0.57) and Asian students (57 %; unadjusted OR 0.61; Table 2 and Appendix 1; Fig. 1). Confounders and socio-demographic characteristics failed to explain these differences (Fig. 1, Appendix 1).

More detailed analysis of the OSCE stations revealed that Dutch students were more likely to pass history taking (84 %) than Surinamese/Antillean students (73 %; unadjusted OR 0.51, 95 % CI 0.31–0.86) and Asian students (76 %; unadjusted OR 0.60, 95 % CI 0.39–0.96). On the physical examination station, Dutch students were more likely to pass (91 %) than Asian students (83 %; unadjusted OR 0.46, 95 % CI 0.27–0.78). On the communication station, Dutch students were more likely to pass (92 %) than Turkish/Moroccan/African students (79 %; unadjusted OR 0.35, 95 % CI 0.21–0.60), Surinamese/Antillean (84 %; unadjusted OR 0.49, 95 % CI 0.26–0.93) and Asian students (82 %; unadjusted OR 0.40, 95 % CI 0.24–0.68).

Dutch students were also more likely to pass the third-year OSCE (79 %) than Turkish/Moroccan/African students (67 %; unadjusted OR 0.54) and Asian students (54 %; unadjusted OR 0.30; Table 2, Appendix 1). Confounders and socio-demographic characteristics could only explain the difference found for the Turkish/Moroccan/African students (Fig. 1, Appendix 1).

More detailed analysis of the stations revealed that Turkish/Moroccan/African students underperformed compared with Dutch students only on physical examination in neurology (pass rate 34 vs 49 %, unadjusted OR 0.55, 95 % CI 0.32–0.94), while Asian students underperformed on history taking (pass rate: 62 vs 78 %; unadjusted OR 0.47, 95 % CI 0.29–0.76), physical examination (pass rate: 83 vs 91 %; unadjusted OR 0.36, 95 % CI 0.21–0.59) and communication (pass rate: 82 vs 92 %; unadjusted OR 0.20, 95 % CI 0.11–0.37). Surinamese/Antillean students only underperformed on the physical examination (pass rate: 67 %; unadjusted OR 0.38, 95 % CI 0.21–0.69), and Western students only on the communication part (pass rate: 88 %; unadjusted OR 0.46, 95 % CI 0.23–0.94).

There were no significant differences in the pass/fail rates between first-generation university students and non-first-generation university students on the OSCEs (Table 3).

## Discussion

Whereas the present study confirms earlier findings of ethnic disparities in preclinical training, it clearly shows that there are differences both across ethnic subgroups and between different types of written and clinical examinations. While all three non-Western ethnic minority groups underperformed on the CPSTs, the language skills test and the OSCEs, findings on the theoretical end-of-block tests and writing skills tests, and results for Western minority students were less consistent. Age, gender, pu-GPA and socio-demographic variables (including parental education and first language) could largely explain the ethnicity-related disparities in theoretical examinations, but not in language, writing and clinical skills examinations. First-generation university students only underperformed on the language skills test.

### Explanations of the findings

One of the most surprising outcomes is the difference in findings on the CPSTs and the end-of-block tests, which are both written theoretical knowledge tests. A possible explanation lies in the nature of, and the required preparation for these examinations. While the end-of-block tests are written tests with series of mostly multiple choice clinical theme-related questions, the CPSTs consist of a number of clinical cases that students have to prepare for in the week preceding the examination. The description of the clinical cases, their elaboration during the preparation for the examination and their subsequent testing in the CPST requires a thorough command of the Dutch language, rendering it probable that these students underperform as a result of a lower level of Dutch language skills. This explanation is confirmed by the fact that the ethnicity-related disparities were further explained after adjusting for the socio-demographic factors including first language.

However, for the preparation of the CPST the involvement of a group of fellow students is essential as well, since the sheer number of differential diagnostic possibilities is much too large for a single student to manage. It might be that the negative effects on learning of ethnic homophily (the tendency to interact with others in the same group) which has been reported in medical students (Vaughan et al. 2015; Woolf et al. 2012) are more profound for this type of examinations. Ethnic homophily may cut off minority students from resources that facilitate learning (Vaughan et al. 2015), which might be particularly important for an examination that requires a high level of self-organised, informal learning.

As previous research showed that all ethnic minority groups, including Western minority students, underperformed in clinical training (Stegers-Jager et al. 2012), it is remarkable that in the current study no underperformance was found in the two OSCEs for Western students. Similarly, the current study also found no indications for a lower level of clinical skills that could explain the previously reported lower clerkship grades for first-generation university students (Stegers-Jager et al. 2012). Apparently, there is a difference in what is measured by the preclinical OSCE stations and the examinations in clinical training. As we have suggested previously (Stegers-Jager et al. 2015), it might be that, due to the more subjective examination methods in clinical training than in preclinical training (Kassebaum and Eaglen 1999), the role of cultural capital (i.e. “knowledge of the norms, styles, conventions and tastes that pervade specific social settings and allow individuals to navigate them in ways that increase their odds of success” (see Massey et al. 2002, p. 6) is more prominent during clinical than during preclinical training.

For the three non-Western ethnic minority groups indications were found for a lower level of clinical skills, and interestingly the different groups underperformed on different parts of the clinical examinations. Our findings were not in line with the study of Fernandez et al. (2007) who found that Asian and Black student only scored lower on the communication part, not on history of physical examination scores. The discrepancy in underperformance of Surinamese/Antillean students on the OSCEs in year 2 and year 3 may be explained by a higher drop-out rate after 2 years at medical school for Surinamese/Antillean students, resulting in a relative loss of the lower performing students from this group (unpublished observation).

Another remarkable finding is that the confounders and socio-demographic factors could largely explain the ethnic related disparities on the theoretical knowledge tests, but to a much lesser extent those on the language, writing and clinical skills examinations. This suggests that the underperformance in the latter examinations is due to other factors, such as cultural differences in communication styles. Hauer et al. (2010) found that lower scores on the communication part of a clinical performance examination for ethnic minority students could partially be explained by a less patient-centred approach. The ethnic minority students scored higher on impersonal attitude, suggesting that they integrate less of the patients’ background and the patients’ perspective into history taking. Another study found that non-native English speakers in Australia scored significantly lower than native English speaking students on appropriate content and appropriate use of the English language for a writing skills assessment (Chur-Hansen and Vernon-Roberts 2000). Nevertheless, further research is required into explanations for the underperformance of ethnic minority students in the clinical and writing skills examinations.

### Comparisons with other studies

Our study confirms that ethnic minority students underperform on written and clinical examinations (Woolf et al. 2011), but also reveals differences in performance among ethnic minority groups and between different types of examinations. In this study we systematically adjusted for a combination of confounders and additional socio-demographic factors, whereas most studies on ethnicity and medical school performance only adjust for gender and sometimes for age, pre-university grades, first language or socio-economic group (Woolf et al. 2011). Our analyses confirmed the expected associations of the confounders with performance at medical school (Appendix 2). The most consistent predictor for underperformance was a missing or lower pu-GPA (Arulampalam et al. 2007; Ferguson et al. 2002; James and Chilvers 2001; Stegers-Jager et al. 2012; Yates and James 2007). In line with other studies (Haq et al. 2005; James and Chilvers 2001; Lumb and Vail 2004; Yates and James 2007), male gender was associated with underperformance in several examinations, but in the end-of-block examination covering biochemistry the male students outperformed the female students. On both types of theoretical knowledge tests the students aged >21 years performed relatively well after adjustment for the other variables (James and Chilvers 2001; Lumb and Vail 2004; Stegers-Jager et al. 2012). The remaining socio-demographic characteristics were less important as predictors of performance in medical school during the pre-clinical years.

### Strengths and limitations

A major strength of this study was its large sample size and hence the large number of non-Dutch students which allowed us to extend our analysis beyond a white/non-white comparison, to which most studies on ethnicity and medical school performance are restricted (Woolf et al. 2011). The use of a longitudinal design, which is also uncommon in studies on factors associated with medical school performance (Ferguson et al. 2002), enabled us to note differences among ethnic groups on several types of pre-clinical examinations. Additionally, unlike previous studies, we were not compelled to use less reliable methods such as self-report or to use names or photographs to gather students’ ethnicity (Haq et al. 2005; Woolf et al. 2011).

There are some limitations to our study. Firstly, our data reveal ethnic disparities in preclinical examinations, but do not shed light on whether these disparities resulted from bias or from actual differences in skills levels on the constructs being assessed. A first strategy to find out whether bias is present is to evaluate the differential predictive validity of examinations or tests. This comprises comparing predicted performance and actual performance, which can either show “underprediction” or “overprediction” (predicted performance lower, respectively higher than actual performance) (Koenig et al. 1998). However, the main challenge for such differential predictive validity studies remains to find a valid, unbiased criterion of medical school performance. A second strategy would be to use differential item functioning procedures to examine for statistical evidence of bias in items (Koenig et al. 1998). Our current study forms a good start for these kinds of follow-up studies.

Secondly, the additional social background data was not collected for all cohorts of students. Therefore we had limited data on the additional socio-demographic factors (urban background, first language, first-generation university student and medical doctor as parent), which were replaced using the multiple imputation technique, a generally accepted and suitable method for dealing with missing values (Donders et al. 2006; Steyerberg 2009). Since they allow the use of data that are available for other predictors that would otherwise be lost, imputation methods, especially multiple imputations, are superior to complete case analysis (Altman and Bland 1995; Donders et al. 2006; Steyerberg 2009). The ORs calculated in the imputed dataset in our study were similar and, if different, generally more conservative than the ORs in the unimputed dataset (Appendix 2), supporting the validity of our use of multiple imputations.

Thirdly, all cohorts of students came from a single medical school. However, there are no reasons to presume that—apart from the relatively large amount of ethnic minority students due to our geographical position in the Netherlands—students at our institution are different from other Dutch medical students with regard to entrance variables (Cohen-Schotanus 1999). Still, replication studies are needed to establish whether the results can be generalised to other populations. We would like to encourage others to also examine ethnic and social disparities in different types of written and clinical examinations.

### Implications for practice and future research

This study has several practical implications for medical schools that are confronted with increasingly diverse student populations. A first practical implication is that medical schools should take care in designing assessment strategies to avoid possible unintended effects of certain types of examinations for certain groups of students. In analogy to the “validity-diversity dilemma” (Kravitz 2008) in selecting for a diverse medical school population, as recently described by Lievens (2015), medical schools face the challenge of balancing assessment strategies that not only fulfil the goal of assessing the required standards of competency, but also retain a diverse student population. In order to ensure that non-traditional medical students are not disadvantaged, diversity should be considered both in test construction and implementation (Wass et al. 2003). Future research should be focused on helping medical schools to design valid assessment strategies that enable non-traditional students to show their merit. As mentioned above, evaluating the differential predictive validity and using differential item functioning procedures may be helpful here.

A second practical implication is that additional support focused on specific examinations for specific groups of students might be appropriate. As an example, the additional support for the CPSTs might take the form of planning formal meetings for students—preferably in randomly allocated tutor groups (Woolf et al. 2012)—to prepare for the examinations. This might lead to ‘meaningful social and academic interactions among students who differ in their experiences, views and traits’ and prevent student from sorting into homogeneous niches (Tienda 2013) which might in particular be disadvantageous for ethnic minority students (Vaughan et al. 2015; Woolf et al. 2012). As stated by Cleland et al. (2013), there is a need for rigorous approaches to developing and evaluating additional support for specific groups, focused on what works and why.

Two additional areas of research within the field of ethnic and social disparities in medical school performance emerge from our findings. Firstly, the previously reported lower clerkship grades for Western minority or first-generation university students (Stegers-Jager et al. 2012) appear not be due to a worse preparation during the pre-clinical years, as both groups of students did not underperform in either the written or the clinical examinations. This was not in line with the findings by Woolf et al. (2008) who found ethnic differences in practical clinical knowledge and skills, but not in theoretical medical knowledge. So, further research is required to explore other causes of the lower grades of Western and first-generation university students in clinical training. Secondly, although the combination of confounders and socio-demographic factors could largely explain the differences in the theoretical examinations, we were still not able to explain the differences in the clinical and writing skills examinations. Despite our own efforts and those of others (Vaughan et al. 2015; Wass et al. 2003; Woolf et al. 2008, 2013), still more research is needed to find explanations for ethnic disparities in the clinical and writing skills examinations.

## Conclusion

Ethnic minority students underperform in pre-clinical training, but there are differences both across ethnic subgroups and between different types of written and clinical examinations. Age, gender and pu-GPA, and socio-demographic variables could largely explain the ethnicity-related disparities in theoretical examinations, but not in language, writing and clinical skills examinations. In order to retain non-traditional students in the medical education pipeline (Lievens 2015), medical schools must design assessment strategies and, if necessary, additional targeted support programmes that create a level playing field for a diverse student population.

## Appendix 1

See Table 4.

**Table 4:** Relationship between ethnicity and performance in written and clinical examinations; Dutch as reference group

Test passed	Ethnicity	OR step 1^a^	95 % CI step 1	OR step 2^b^	95 % CI step 2	OR step 3^c^	95 % CI step 3
CPST year 1 (n = 1837)	T/M/A	**0.52**	0.36–0.74	0.71	0.48–1.05	0.88	0.46–1.70
	S/A	**0.46**	0.31–0.68	0.67	0.43–1.04	0.77	0.47–1.26
	Asian	**0.46**	0.33–0.64	**0.52**	0.36–0.75	0.71	0.37–1.38
	Western	0.97	0.66–1.42	0.99	0.65–1.49	1.16	0.67–2.01
CPST year 2 (n = 1278)	T/M/A	**0.53**	0.33–0.83	0.75	0.46–1.22	0.99	0.40–2.46
	S/A	**0.38**	0.24–0.62	**0.47**	0.28–0.80	**0.50**	0.27–0.91
	Asian	**0.47**	0.31–0.71	**0.58**	0.37–0.92	0.66	0.26–1.69
	Western	0.82	0.52–1.30	0.86	0.53–1.41	0.94	0.50–1.75
CPST year 3 (n = 963)	T/M/A	**0.43**	0.24–0.75	**0.55**	0.30–0.99	0.88	0.32–2.43
	S/A	0.68	0.35–1.34	0.87	0.42–1.79	1.11	0.47–2.64
	Asian	**0.29**	0.18–0.46	**0.30**	0.18–0.51	0.47	0.18–1.24
	Western	**0.48**	0.28–0.80	**0.42**	0.24–0.73	0.53	0.27–1.05
Block test year 1 A (n = 1902)	T/M/A	0.72	0.51–1.02	1.02	0.70–1.49	1.17	0.67–2.05
	S/A	**0.59**	0.40–0.87	0.93	0.61–1.42	0.95	0.60–1.51
	Asian	0.81	0.58–1.13	1.04	0.72–1.50	1.11	0.60–2.07
	Western	0.87	0.62–1.23	0.88	0.60–1.28	0.93	0.58–1.47
Block test year 1 B (n = 1884)	T/M/A	**0.59**	0.42–0.85	0.87	0.59–1.29	0.83	0.40–1.75
	S/A	**0.63**	0.42–0.95	1.03	0.66–1.62	0.95	0.57–1.60
	Asian	0.72	0.50–1.03	0.95	0.64–1.41	0.83	0.37–1.83
	Western	**1.92**	1.20–3.07	**2.19**	1.33–3.61	**2.13**	1.11–4.07
Language skills test (n = 2233)	T/M/A	**0.36**	0.26–0.50	**0.48**	0.33–0.68	**0.52**	0.33–0.83
	S/A	**0.46**	0.32–0.66	0.80	0.53–1.19	0.90	0.59–1.37
	Asian	**0.30**	0.22–0.42	**0.34**	0.24–0.49	**0.44**	0.26–0.74
	Western	**0.72**	0.53–0.97	0.78	0.56–1.09	0.86	0.59–1.25
Writing skills test year 1 (n = 1519)	T/M/A	**0.56**	0.38–0.84	0.74	0.49–1.14	0.57	0.29–1.12
	S/A	0.63	0.39–1.01	0.79	0.48–1.32	0.82	0.47–1.45
	Asian	**0.67**	0.45–0.99	0.78	0.52–1.19	0.84	0.41–1.75
	Western	1.06	0.67–1.68	1.12	0.69–1.81	1.13	0.66–1.94
Writing skills test year 2 (n = 1020)	T/M/A	**0.44**	0.23–0.82	**0.50**	0.26–0.96	0.49	0.20–1.18
	S/A	0.84	0.35–2.03	1.05	0.41–2.67	1.14	0.41–3.16
	Asian	0.54	0.29–1.01	0.60	0.31–1.16	0.68	0.24–1.92
	Western	1.28	0.54–3.05	1.59	0.65–3.89	1.70	0.63–4.59
Writing skills test year 3 (n = 949)	T/M/A	**0.50**	0.30–0.84	0.59	0.35–1.02	0.57	0.21–1.53
	S/A	0.60	0.34–1.06	0.77	0.42–1.41	0.77	0.37–1.58
	Asian	**0.47**	0.30–0.76	**0.52**	0.32–0.86	0.42	0.17–1.07
	Western	0.80	0.49–1.29	0.72	0.44–1.20	0.68	0.36–1.27
OSCE year 2 (n = 1379)	T/M/A	**0.55**	0.37–0.84	**0.60**	0.39–0.92	0.57	0.28–1.14
	S/A	**0.57**	0.36–0.90	**0.57**	0.35–0.92	**0.57**	0.33–0.99
	Asian	**0.53**	0.36–0.78	**0.53**	0.35–0.79	0.52	0.25–1.07
	Western	0.96	0.64–1.44	0.94	0.62–1.42	0.90	0.54–1.50
OSCE year 3 (n = 962)	T/M/A	**0.54**	0.31–0.93	0.63	0.36–1.11	0.84	0.31–2.25
	S/A	0.58	0.32–1.05	0.73	0.39–1.36	0.91	0.45–1.84
	Asian	**0.30**	0.19–0.49	**0.32**	0.20–0.53	**0.35**	0.14–0.90
	Western	0.75	0.45–1.25	0.77	0.46–1.30	0.83	0.43–1.57

*OR* odds ratio, *95* *% CI* 95 % confidence interval, *T/M/A* Turkish/Moroccan/African, *S/A* Surinamese/Antillean, *CPST* Clinical Problem Solving Test, *OSCE* Objective Structured Clinical Examination

Figures in bold denote significant odds ratios (*p* < 0.05)

^a^Step 1: ethnicity effect unadjusted

^b^Step 2: ethnicity effect adjusted for age, gender and pre-university grade point average (confounders) and cohort

^c^Step 3 ethnicity effect adjusted for confounders, cohort and first-generation immigrant, urban background, first language non-Dutch, medical doctor as parent and first-generation university (socio-demographic characteristics)

## Appendix 2

See Tables 5 and 6.

**Table 5:** Detailed description of the multivariable models to adjust the relationship of ethnicity and social background with performance in the theoretical knowledge examinations

	CPST year 1		CPST year 2		CPST year 3		Block test year 1 A		Block test year 1 B	
	MI (n = 1837) OR (95 % CI)	CC (n = 629) OR (95 % CI)	MI (n = 1317) OR (95 % CI)	CC (n = 327) OR (95 % CI)	MI (n = 974) OR (95 % CI)	CC (n = 323) OR (95 % CI)	MI (n = 1895) OR (95 % CI)	CC (n = 643) OR(95 % CI)	MI (n = 1884) OR(95 % CI)	CC (n = 638) OR (95 % CI)
*Ethnicity*										
Dutch	1.00	1.00	1.00	1.00	1.00	1.00	1.00	1.00	1.00	1.00
T/M/A	0.88 (0.46–1.70)	0.88 (0.35–2.24)	0.99 (0.40–2.46)	1.68 (0.32–8.73)	0.88 (0.32–2.43)	0.93 (0.13–6.44)	1.17 (0.67–2.05)	1.28 (0.50–3.28)	0.83 (0.40–1.75)	0.48 (0.17–1.31)
S/A	0.77 (0.47–1.26)	0.90 (0.36–2.27)	**0.50** **(0.27–0.91)**	0.86 (0.22–3.35)	1.11 (0.47–2.64)	0.71 (0.12–4.34)	0.95 (0.60–1.51)	1.27 (0.49–3.30)	0.95 (0.57–1.60)	1.35 (0.44–4.14)
Asian	0.71 (0.37–1.38)	1.24 (0.45–3.47)	0.66 (0.26–1.69)	2.33 (0.46–11.91)	0.47 (0.18–1.24)	0.30 (0.05–1.83)	1.11 (0.60–2.07)	1.47 (0.53–4.10)	0.83 (0.37–1.83)	0.56 (0.18–1.75)
Western	1.16 (0.67–2.01)	1.00 (0.45–2.28)	0.94 (0.50–1.75)	1.60 (0.44–5.75)	0.53 (0.27–1.05)	0.43 (0.10–1.82)	0.93 (0.58–1.47)	1.16 (0.52–2.59)	**2.13** **(1.11–4.07)**	3.11 (0.84–11.48)
Male	0.83 (0.66–1.04)	0.99 (0.67–1.48)	**0.71** **(0.53–0.95)**	0.62 (0.34–1.12)	0.96 (0.67–1.38)	1.09 (0.49–2.43)	**1.27** **(1.02–1.58)**	1.41 (0.96–2.09)	0.99 (0.78–1.25)	0.74 (0.47–1.15)
*Age*										
<19	1.00	1.00	1.00	1.00	1.00	1.00	1.00	1.00	1.00	1.00
19–21	0.84 (0.66–1.07)	1.15 (0.75–1.75)	0.74 (0.55–1.00)	0.71 (0.36–1.37)	0.98 (0.67–1.45)	0.90 (0.38–2.11)	0.92 (0.73–1.16)	1.10 (0.73–1.65)	0.89 (0.69–1.15)	1.18 (0.73–1.91)
>21	**2.07** **(1.40–3.05)**	**2.80** **(1.43–5.49)**	**1.67** **(1.03–2.71)**	2.00 (0.74–5.44)	1.58 (0.86–2.88)	2.56 (0.74–8.85)	**1.81** **(1.27–2.59**)	**2.49** **(1.32–4.72)**	1.45 (0.99–2.12)	1.84 (0.91–3.72)
*pu*-*GPA* ^*a*^										
Missing	**0.25** **(0.17–0.35)**	**0.25** **(0.13–0.51)**	**0.47** **(0.29–0.77)**	0.42 (0.15–1.19)	0.78 (0.42–1.44)	0.30 (0.08–1.12)	**0.29** **(0.20–0.41)**	0.52 (0.26–1.04)	**0.25** **(0.18–0.37)**	**0.26** **(0.13–0.54)**
Continuous	**4.59** **(3.58–5.89)**	**6.29** **(4.04–9.78)**	**3.91** **(2.88–5.30)**	**3.71** **(1.95–7.07)**	**2.85** **(1.98–4.12)**	**10.70** **(3.91–29.28)**	**5.39** **(4.26–6.83)**	**5.81** **(3.84–8.79)**	**5.96** **(4.54–7.83)**	**6.26** **(3.78–10.37)**
First-generation immigrant	0.83 (0.51–1.28)	0.75 (0.32–1.79)	1.36 (0.73–2.54)	0.63 (0.19–2.08)	1.00 (0.50–1.99)	1.78 (0.46–6.91)	1.13 (0.71–1.79)	0.77 (0.33–1.81)	1.17 (0.70–1.93)	0.65 (0.24–1.75)
Urban background	0.84 (0.62–1.13)	0.69 (0.46–1.04)	0.94 (0.65–1.37)	0.58 (0.31–1.10)	0.79 (0.45–1.40)	0.47 (0.20–1.10)	0.93 (0.65–1.33)	0.76 (0.52–1.12)	0.96 (0.69–1.33)	1.06 (0.68–1.67)
First language non-Dutch	0.83 (0.44–1.54)	0.70 (0.32–1.51)	0.71 (0.23–2.14)	0.40 (0.11–1.45)	0.62 (0.22–1.74)	0.63 (0.13–3.06)	0.86 (0.44–1.70)	0.63 (0.29–1.37)	1.07 (0.43–2.63)	1.71 (0.69–4.26)
Medical doctor as parent	0.99 (0.68–1.43)	1.05 (0.56–1.98)	0.86 (0.49–1.53)	0.89 (0.32–2.45)	0.76 (0.40–1.42)	0.95 (0.23–3.92)	1.01 (0.72–1.41)	1.28 (0.69–2.37)	1.05 (0.69–1.58)	1.27 (0.61–2.64)
First-generation university	1.09 (0.68–1.74)	1.26 (0.78–2.04)	1.12 (0.62–2.02)	1.36 (0.65–2.83)	1.02 (0.51–2.03)	1.11 (0.45–2.74)	1.02 (0.63–1.65)	1.25 (0.79–1.97)	0.97 (0.62–1.51)	1.29 (0.76–2.19)

*CPST* Clinical Problem Solving Test, *MI* multiple imputed, *CC* complete cases, *OR* odds ratio, *95* *% CI* 95 % confidence interval, *T/M/A* Turkish/Moroccan/African, *S/A* Surinamese/Antillean, *pu*-*GPA* pre-university grade point average

Figures in bold denote significant odds ratios (*p* < 0.05)

^a^Missing values for pre-university GPA were substituted to the mean with Missing pu-GPA and continuous pu-GPA included

**Table 6:** Detailed description of the multivariable models to adjust the relationship of ethnicity and social background with performance in the language, writing and clinical skills examinations

	Language skills test		Writing skills test year 1		Writing skills test year 2		Writing skills test year 3	
	MI (n = 2233) OR (95 % CI)	CC (n = 958) OR (95 % CI)	MI (n = 1519) OR (95 % CI)	CC (n = 638) OR (95 % CI)	MI(n = 1020) OR (95 % CI)	CC (n = 321) OR (95 % CI)	MI(n = 949) OR (95 % CI)	CC (n = 312) OR (95 % CI)
*Ethnicity*								
Dutch	1.00	1.00	1.00	1.00	1.00	1.00	1.00	1.00
T/M/A	**0.52** **(0.33–0.83)**	0.60 (0.29–1.27)	0.57 (0.29–1.12)	0.44 (0.18–1.10)	0.49 (0.20–1.18)	0.22 (0.04–1.41)	0.57 (0.21–1.53)	0.28 (0.06–1.31)
S/A	0.90 (0.59–1.37)	1.29 (0.63–2.64)	0.82 (0.47–1.45)	1.25 (0.47–3.32)	1.14 (0.41–3.16)	0.38 (0.07–2.06)	0.77 (0.37–1.58)	1.32 (0.34–5.20)
Asian	**0.44** **(0.26–0.74)**	0.67 (0.32–1.43)	0.84 (0.41–1.75)	0.56 (0.21–1.49)	0.68 (0.24–1.92)	0.92 (0.12–7.38)	0.42 (0.17–1.07)	0.87 (0.21–3.73)
Western	0.86 (0.59–1.25)	1.06 (0.60–1.90)	1.13 (0.66–1.94)	1.23 (0.52–2.87)	1.70 (0.63–4.59)	1.70 (0.26–11.07)	0.68 (0.36–1.27)	0.89 (0.32–2.44)
Male	0.87 (0.72–1.06)	0.80 (0.59–1.07)	**0.75** **(0.58–0.96)**	0.77 (0.52–1.13)	**0.62** **(0.41–0.95)**	1.07 (0.44–2.60)	1.07 (0.79–1.46)	1.05 (0.61–1.80)
*Age*								
<19	1.00	1.00	1.00	1.00	1.00	1.00	1.00	1.00
19–21	0.93 (0.75–1.15)	0.90 (0.66–1.23)	1.13 (0.86–1.49)	**1.77** **(1.15–2.72)**	1.21 (0.75–1.95)	1.07 (0.39–2.94)	1.01 (0.72–1.40)	1.06 (0.59–1.88)
>21	0.78 (0.55–1.10)	0.77 (0.46–1.31)	1.20 (0.80–1.81)	1.64 (0.88–3.07)	0.95 (0.47–1.89)	0.52 (0.15–1.85)	1.51 (0.90–2.53)	1.58 (0.66–3.76)
*pu-GPA*								
Missing	**0.24** **(0.17–0.34)**	**0.16** **(0.08–0.30)**	**0.39** **(0.26–0.59)**	**0.35** **(0.18–0.69)**	**0.43** **(0.22–0.85)**	0.40 (0.11–1.44)	**0.50** **(0.30–0.84)**	0.46 (0.17–1.24)
Continuous	**3.36** **(2.79–4.03)**	**2.93** **(2.24–3.83**)	**3.26** **(2.50–4.26)**	**3.36** **(2.28–4.97)**	**1.81** **(1.18–2.78)**	1.46 (0.61–3.47)	**2.48** **(1.84–3.35)**	**2.09** **(1.27–3.42)**
First-generation immigrant	0.65 (0.42–1.01)	0.68 (0.34–1.36)	**0.59** **(0.34–1.01)**	0.77 (0.32–1.80)	0.76 (0.32–1.76)	0.31 (0.07–1.38)	0.99 (0.50–1.96)	0.35 (0.11–1.10)
Urban background	0.95 (0.77–1.18)	0.92 (0.69–1.23)	1.22 (0.91–1.63)	1.38 (0.93–2.05)	0.92 (0.52–1.62)	1.01 (0.41–2.48)	0.71 (0.40–1.27)	0.64 (0.37–1.10)
First language non-Dutch	0.98 (0.62–1.54)	1.02 (0.56–1.89)	1.28 (0.61–2.71)	1.51 (0.69–3.30)	1.10 (0.49–2.44)	1.22 (0.26–5.80)	1.42 (0.51–3.96)	1.83 (0.55–6.09)
Medical doctor as parent	0.80 (0.60–1.07)	0.83 (0.53–1.29)	1.09 (0.61–1.95)	0.93 (0.51–1.70)	1.06 (0.58–1.97)	1.14 (0.23–5.66)	0.79 (0.42–1.48)	0.73 (0.31–1.73)
First-generation university	0.91 (0.69–1.19)	0.79 (0.56–1.11)	1.08 (0.78–1.50)	1.13 (0.72–1.79)	1.01 (0.53–1.94)	0.96 (0.35–2.59)	0.79 (0.44–1.41)	0.76 (0.41–1.39)

	OSCE year 2		OSCE year 3	
	MI (n = 1379) OR (95 % CI)	CC (n = 331) OR (95 % CI)	MI (n = 962) OR (95 % CI)	CC (n = 320) OR (95 % CI)
*Ethnicity*				
Dutch	1.00	1.00	1.00	1.00
T/M/A	0.57 (0.28–1.14)	1.37 (0.27–7.02)	0.84 (0.31–2.25)	2.53 (0.38–17.01)
S/A	**0.57** **(0.33–0.99)**	0.48 (0.14–1.73)	0.91 (0.45–1.84)	3.40 (0.56–20.51)
Asian	0.52 (0.25–1.07)	0.58 (0.14–2.31)	**0.35** **(0.14–0.90)**	0.88 (0.19–4.14)
Western	0.90 (0.54–1.50)	0.53 (0.19–1.46)	0.83 (0.43–1.57)	0.60 (0.21–1.76)
Male	**0.58** **(0.46–0.74)**	**0.49** **(0.29–0.86)**	0.86 (0.62–1.19)	0.67 (0.36–1.22)
*Age*				
<19	1.00	1.00	1.00	1.00
19–21	1.15 (0.88–1.51)	**1.91** **(1.01–3.60)**	1.18 (0.82–1.68)	1.48 (0.75–2.92)
>21	1.24 (0.81–1.88)	1.55 (0.62–3.85)	1.22 (0.71–2.09)	2.44 (0.90–6.66)
*pu-GPA* ^a^				
Missing	1.14 (0.74–1.76)	1.35 (0.45–4.10)	0.65 (0.36–1.16)	**0.27** **(0.09–0.78)**
Continuous	**1.55** **(1.23–1.94)**	1.54 (0.94–2.55)	**1.79** **(1.31–2.47)**	**2.76** **(1.52–5.03)**
First-generation immigrant	0.85 (0.50–1.43)	0.74 (0.25–2.16)	0.80 (0.40–1.59)	1.08 (0.31–3.80)
Urban background	0.94 (0.63–1.41)	0.71 (0.40–1.27)	**0.47** **(0.26–0.85)**	**0.40** **(0.21–0.77)**
First language non-Dutch	1.17 (0.56–2.44)	0.77 (0.25–2.41)	1.23 (0.36–4.19)	0.96 (0.27–3.49)
Medical doctor as parent	1.01 (0.53–1.93)	2.20 (0.69–6.98)	0.85 (0.48–1.53)	0.80 (0.29–2.21)
First-generation university	0.93 (0.64–1.34)	0.79 (0.42–1.46)	0.79 (0.41–1.51)	0.60 (0.31–1.19)

*OSCE* Objective Structured Clinical Examination, *MI* multiple imputed, *CC* complete cases, *OR* odds ratio, *95* *% CI* 95 % confidence interval, *T/M/A* Turkish/Moroccan/African, *S/A* Surinamese/Antillean, *pu*-*GPA* pre-university grade point average

Figures in bold denote significant odds ratios (*p* < 0.05)

^a^Missing values for pre-university GPA were substituted to the mean with Missing pu-GPA and continuous pu-GPA included
